# Infection and seroprevalence of *Borrelia persica* in domestic cats and dogs in Israel

**DOI:** 10.1186/s13071-022-05223-9

**Published:** 2022-05-10

**Authors:** Gad Baneth, Ann Dvorkin, Bar Ben-Shitrit, Gabriela Kleinerman, Harold Salant, Reinhard K. Straubinger, Yaarit Nachum-Biala

**Affiliations:** 1 The Koret School of Veterinary Medicine, The Hebrew University of Jerusalem, P.O. Box 12, 761001 Rehovot, Israel; 2 Bacteriology and Mycology, Institute for Infectious Diseases and Zoonoses, Ludwig-Maximilians-Universität München, Munich, Germany

**Keywords:** Anemia, *Borrelia persica*, Cat, Dog, Israel, Thrombocytopenia, Tick-borne relapsing fever

## Abstract

**Background:**

Relapsing fever borreliosis is an infectious disease caused by bacteria of the genus *Borrelia,* inflicting recurrent episodes of fever and spirochetemia in humans. *Borrelia persica,* the causative agent of relapsing fever in Israel, is prevalent over a broad geographic area that extends from India to Egypt. It is transmitted by the soft tick *Ornithodoros tholozani* and causes disease in humans as well as domestic cats and dogs. The goal of this study was to survey domestic dogs and cats in Israel for infection with *B. persica*.

**Methods:**

Blood, sera and demographic and clinical data were collected from dogs and cats brought for veterinary care in central Israel. PCR followed by DNA sequencing was used to detect *B. persica* DNA in blood samples, and an enzyme-linked immunosorbent assay (ELISA) was used to detect antibodies reactive with *B. persica* antigens in sera from the same animals. This is the first serological survey of *B. persica* in dogs and the first survey for antibodies reactive with a relapsing fever *Borrelia* sp. in cats globally.

**Results:**

Four of the 208 dogs (1.9%) and three of 103 cats (2.9%) sampled were positive by PCR for *B. persica* DNA, and 24 dogs (11.5%) and 18 cats (17.5%) were seropositive for *B. persica* antigen by ELISA. The ratio between PCR-positivity and seropositivity in both the dog and cat populations was 1:6. All four PCR-positive dogs and two of three PCR-positive cats were seronegative, suggesting a probable recent infection. Thrombocytopenia showed significant association with seropositivity in dogs (*P* = 0.003). In cats, anemia had a significant association with seropositivity (*P* = 0.0001), and thrombocytopenia was associated with the combined prevalence of seropositivity or PCR-positivity (*P* = 0.022).

**Conclusions:**

*Borrelia persica* infection is more prevalent and widespread in domestic canine and feline populations in Israel than previously thought. Dogs and cats may play a role as reservoirs and sentinels for human infection. Precautions should be taken to prevent transfusion-transmitted infection between blood donor and recipient animals.

**Graphic Abstract:**

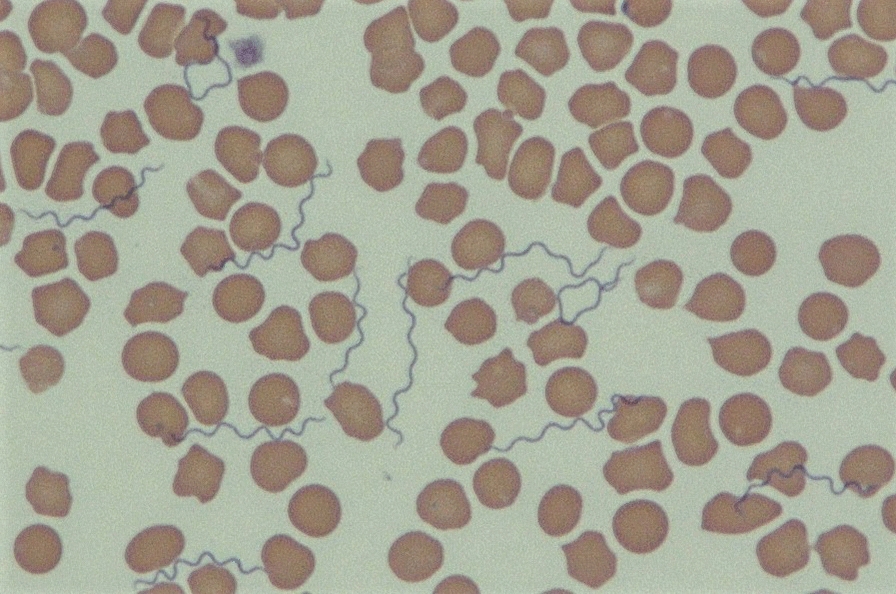

## Background

Tick-borne relapsing fever (TBRF) is an acute infectious disease characterized in humans by recurrent febrile episodes that concur with spirochetemia. The causative agents of TBRF are classified in the order Spirochaetales and belong to the genus *Borrelia* [1]. The disease incubation period in humans is 2–12 days, followed by the appearance of fever, chills, headache, myalgia, arthralgia and abdominal pain. Fatal infections are associated with complications such as myocarditis, nephritis, liver failure and cerebral hemorrhage [2]. *Borrelia persica*, the causative agent of TBRF in parts of central Asia, the Middle East and the eastern Mediterranean region including Israel, is transmitted via the bite of the soft tick *Ornithodoros tholozani* [3]. *Ornithodoros tholozani* has been reported in India, central Asian states, including Kazakhstan, Kirgizstan, Tajikistan, Turkmenistan and Uzbekistan, and Iran, Iraq, Syria, Jordan, Turkey and Egypt [4]. It inhabits dark, warm, moist spaces (humidity: 70–80%; temperature range: 17–25 °C), such as caves and ruins where it burrows under clods of earth or hides in cracks [5, 6].

TBRF was described in Israel in the 1920s [7] and is often referred to as “cave fever” since caves and abandoned buildings are common locations for acquiring infection. In three different studies, 64, 71 and 83%, respectively, of the patients diagnosed with TBRF reported visiting a cave prior to developing disease symptoms [2, 8, 9]. According to reports of the Epidemiological Department of the Israeli Ministry of Health, the incidence of TBRF in the Israeli population ranges from 1 to 3 cases per 100,000 people annually [2]. The highest incidence of clinical cases of TBRF in Israel is reported among military personnel: between 1982 and 2003, the incidence was 6.3 cases per 100,000 soldiers, while among the civilian population the prevalence declined from 0.35 to 0.11 cases per 100,000 people during this same period [2].

*Borrelia persica* infection accompanied by high spirochetemia levels and clinical disease was reported in domestic dogs and cats in Israel in 2016 [10]. Additional studies conducted in Israel using PCR detected *B. persica* infection in golden jackals (*Canis aureus*), red foxes (*Vulpes vulpes*), Indian crested porcupines (*Hystrix indica*), rock hyraxes (*Procavia capensis*), Cairo spiny mice (*Acomys cahirinus*) and other wildlife mammals [11, 12]. Wildlife canids were recognized as the main reservoirs for tick infection based on blood meal analysis [12]. Other relapsing fever *Borrelia* spp., such as *Borrelia turicatae* and *Borrelia hermsii*, have been reported to cause disease in dogs in North America [13–15], and *Borrelia hispanica* was associated with disease in a dog in Europe [16]. *Borrelia hispanica* and *Borrelia miyamotoi* have been reported to infect cats in Europe and North America, respectively [16, 17].

 The aim of this study was to survey populations of dogs and cats in Israel for infection with *B. persica*. This is the first serological survey of *B. persica* in dogs and the first survey for antibodies reactive with relapsing fever *Borrelia* spp. in cats.

## Methods

### Samples collection

Canine and feline whole blood samples anti-coagulated with EDTA and sera samples from the same animals were collected between 2017 and 2019 from domestic cats and dogs that received medical care at the Hebrew University Veterinary Teaching Hospital in Beit Dagan and from private veterinary clinics in the cities of Rehovot, Kfar Saba, Ma’ale Adumim and Jerusalem in Israel**.**

Recorded data obtained from the animals’ medical records included sex, breed, age group (juvenile or older than 1 year), previous antibiotic treatment up to 3 months prior to the blood collection, rural or urban setting (rural settlement was defined as an isolated locality with < 2000 residents), climate (Mediterranean, semi-arid and arid), residence location and geographical area of residence. The residence location referred to the animals' permanent accommodation address. Geographic areas of residence included five regions: central Israel (an inland region east of the coastal plain in Israel), Jerusalem (the city of Jerusalem and its suburbs), Judean desert (a desert region east of Jerusalem extending to the Dead Sea), coastal plain (a plain region bordering the Mediterranean around Tel Aviv) and south-central Israel (a region south of Tel Aviv and north of the city of Beer Sheva).

The clinical variables examined included physical examination findings described in *B. persica* infection in dogs and cats, including fever, lethargy, bleeding, inappetence and icterus visible on physical examination, or total bilirubin > 2 mg/dL in the animal's serum biochemistry results [10]. Additional clinical parameters included anemia (defined as red blood cell count < 6 × 10^6^ cells/μl for cats and dogs), hematocrit < 30% for dogs and 25% for cats and thrombocytopenia (defined as a platelet count < 140,000/μl for dogs and < 150,000/μl for cats).

This study was approved by the Internal Research Committee of the Koret School of Veterinary Medicine Veterinary Teaching Hospital 2017 (KSVM-VTH/12_2017) and included residual samples from blood collected for routine testing as a part of the animal’s diagnostic procedures.

### Molecular identification and characterization of *B. persica*

DNA was extracted from a 250-μl sample of whole blood using the Illustra blood genomicPrep Mini Spin Kit (GE Health care, Chicago, IL, USA), following the manufacturer’s instructions. Canine and feline blood samples were tested for infection with *B. persica* by real-time PCR using the *flaB* gene as a target. Real-time PCR was conducted using primers Bfpbu (5′-GCT GAA GAG CTT GGA ATG CAA CC-3′) and Bfpcr (5′-TGA TCA GTT ATC ATT CTA ATA GCA-3′) for the amplification of a 346-bp fragment of the *flaB* gene of *Borrelia* spp. [18], in the StepOnePlus real-time PCR thermal cycler (Applied Biosystems, Thermo Fisher Scientific, Foster City, CA, USA). The real-time PCR cycling conditions for the *flaB* gene were: an initial hold for 4 min at 95 °C, followed by 45 cycles of 15 s at 95 °C, 30 s at 60 °C and 10 s at 72 °C; the melting phase started at 60 °C, with each step increasing by 0.3 °C, and finished at 95 °C with a hold for 90 s at the first step and 5 s at subsequent steps. Each reaction was performed in a total reaction volume of 20 μl containing 4 μl of DNA solution, 0.5 μM of each primer, 0.6 μl of Syto9 (Invitrogen, Thermo Fisher Scientific, Carlsbad, CA, USA), 4.4 μl of ultra-pure water (Biological Industries, Beit Haemek, Israel) and 10 μl of Maxima Hot Start PCR Master Mix (Thermo Fisher Scientific, Waltham, MA, USA). DNA from plasmids containing the DNA of *flaB* fragments (1 μl DNA) was used as positive controls and DNA from a *Borrelia*–negative dog and a non-template control were run with each reaction. PCR-positive products were sequenced to verify the identity of the infecting *Borrelia* spp. The sequencing was performed at the Center for Genomic Analyses at the Hebrew University (Jerusalem, Israel) using the BigDye Terminator Cycle Sequencing Kit (Applied Biosystems ABI3700 DNA analyzer) and ABI Data Collection and Sequence Analysis software (Applied Biosystems, Thermo Fisher Scientific, Foster City, CA, USA). DNA sequences were compared for similarity to other sequences in GenBank using the BLASTn program hosted by NCBI, National Institutes of Health, USA (http://www.ncbi.nlm.nih.gov).

### Enzyme-linked immunosorbent assay for specific antibodies in dogs reactive with *B. persica* antigens

Serum samples from the same dogs from which whole blood had been collected and subjected to PCR were obtained and analyzed by enzyme-linked immunosorbent assay (ELISA) for antibodies reactive with *B. persica* antigen. All serum samples were stored at − 20 °C prior to testing. *Borrelia persica* antigen isolated and grown in culture from an Israeli cat was used [19]. ELISA plates containing 96 wells were coated for 16 h at 4 °C with 6.25 ng *B. persica*-specific antigen dissolved in phosphate-buffered saline (PBS). After three successive washes with PBS containing 0.1% Tween-20, blocking was performed with 2% fetal bovine serum (FBS; Biological Industries) in PBS overnight at 4 °C. Serum samples from a dog diagnosed with *B. persica* by blood smear microscopy and verified by PCR and sequencing was used as a positive control, and serum from a colony bred dog with no exposure to ticks was used as a negative control [10]. The plates were washed three times, as mentioned above, and the tested sera diluted at 1:500 in PBS with 0.1% Tween 20 and 2% FBS and incubated with *B. persica* antigen-coated plates for 1 h at 37 °C. After three successive washes, the remaining bound antibodies were incubated for 1 h at 37 °C with horseradish peroxide (HRP)–conjugated protein A (Zymed Laboratories, Inc., San Francisco, CA, USA) and diluted at 1:10,000 in PBS with 0.1% Tween 20 and 2% FBS. Excess conjugate was removed by washing as described above, and a colorimetric reaction was carried out by addition of the chromogen 2,2'-azino-bis (3–ehtylbenzthiazoline-6-sulfonic acid) ammonium salt (ABTS) (Boehringer Mannheim, Ingelheim, Germany) mixed in ABTS buffer. Each plate was read when the absorbance (λ = 405 nm) of the positive reference serum reached an optical density (OD) value between 1.2 and 1.4. The OD readings were standardized to minimize inter-plate variation by using the same positive control on every plate and adjusting to a constant positive control value. A cut-off value of 0.23 OD was calculated by adding three standard deviations to the mean absorbance of sera from 12 research breeding colony dogs [20]. All samples above the cut-off were considered to be positive for the statistical analyses.

### ELISA for specific antibodies in cats reactive with *B. persica* antigens

ELISA plate preparation and antigen coating for the feline ELISA were performed as described in the preceding section for detection of antibodies reactive with *B. persica* in dogs. Sera from four naturally infected *B. persica* PCR-positive cats with clinical disease were used to initially evaluate the serologic response to *B. persica* antigen [10]. Serum dilutions were tested at 1:50, 1:100 and 1:500, with the sera diluted in PBS with 0.1% Tween 20 and 2% FBS. The diluted sera were incubated with *B. persica* antigen-coated plates for 1 h at 37 °C, following which the plates were washed with 0.1% Tween 20 in 50 mM PBS at pH 7.2. After three successive washes, the remaining bound antibodies were incubated for 1 h at 37 °C with rabbit anti-cat IgG antigen conjugated to HRP as secondary antibody (OriGene Technologies GmbH, Herford Innenstadt, Germany). In addition, the ability of positive reference serum absorbance (λ = 405 nm) to reach an OD value of between 1.1 and 1.2 was tested by applying different anti-feline IgG dilutions (1:5000 and 1:10,000.) The serum dilution of 1:500 was subsequently chosen as the optimal dilution that provided the best discrimination between positive and negative samples, and anti-feline IgG used as secondary antibody was diluted 1:10,000 according to the manufacturer's instructions. A serological cut-off of 0.29 OD for cats was calculated based on three standard deviations above the mean OD values of readings from the sera of eight *B. persica* PCR-negative cats from non-endemic areas for borreliosis. All samples above the cut-off value were considered as seropositive.

### Statistical analysis

Statistical analysis was performed using the statistical software program SPSS version 25.0 (SPSS IBM, Armonk, NY, USA). Statistical significance was defined as *P* < 0.05. The Chi-square test was used to test the potential impact of different variables on an outcome. The continuity correction was employed when 2 × 2 tables were used. Sample size was calculated using WinPepi software version 11.65 (http://www.brixtonhealth.com/pepi4windows.html). Assuming the prevalence of infection among domestic canines and felines in Israel resembles the prevalence among the golden jackal population in Israel, which was 1% (G Baneth, unpublished data), we calculated that at least 80 dogs and 80 cats should be sampled with a 95% confidence interval at an accepted difference of ± 0.1.

## Results

Samples were collected from a total of 208 dogs and 103 cats. Of these, four of the dogs (1.9%) and three of the cats (2.9%) were positive by PCR for *B. persica* DNA, and 24 dogs (11.5%) and 18 cats (17.5%) had seropositive responses to *B. persica* antigen by ELISA (Tables 1, 2).

**Table 1:** Prevalence of exposure and infection with *Borrelia persica* and demographic characteristics in 208 dogs as determined by DNA detection by PCR and antibody detection by ELISA

Variable	Number of dogs	PCR+ (%)	Seropositive (%)^a^	PCR+ or seropositive (%)^b^
Total	208	4 (1.9)	24 (11.5)	28 (11.5)
*Sex*				
Female	99	3 (2.8)	12 (11)	15 (13.7)
Male	109	1 (1)	12 (12.1)	13 (13.1)
Statistical significance			*χ*^2^ = 0.001, *df* = 1,* P* = 0.973	*χ*^2^ = 0.001, *df* = 1, *P* = 0.973
*Breed*				
Purebred	76	3 (3.9)	9 (11.8)	12 (15.78)
Mixed	132	1 (9.1)	15 (11.3)	16 (12.1)
Statistical significance			*χ*^2^ = 0.0001, *df* = 1,* P* = 1	*χ*^2^ = 0.287, *df* = 1, *P* = 0.592
*Age*				
Juvenile	9	0 (0)	1 (11.1)	1 (11.1)
Adult	199	4 (2.1)	23 (11.5)	27 (13.5)
Statistical significance			*χ*^2^ = 0.0001, *df* = 1,* P* = 1	*χ*^2^ = 0.0001, *df* = 1, *P* = 1
*Antibiotic treatment*				
Yes	16	0 (0)	0 (0)	0 (0)
No	108	3 (2.7)	19 (17.6)	22 (20)
Unknown	84	1 (1.1)	5 (5.9)	6 (7.14)
Statistical significance			*χ^2^ = 8.533,* df* = 2,* P* = 0.014	*χ^2^ = 9.793,* df* = 2, *P* = 0.007
*Habitat*				
Urban	183	4 (2.3)	20 (10.9)	24 (13.1)
Rural	25	0 (0)	4 (16)	4 (16)
Statistical significance			*χ*^2^ = 0.169, *df* = 1,* P* = 0.681	*χ*^2^ = 0.007, *df* = 1, *P* = 0.933
*Climate*				
Mediterranean	143	3 (2)	13 (9)	16 (11.1)
Semi-arid	65	1 (1.5)	11 (17)	12 (18.4)
Arid	0	0	0	0
Statistical significance			*χ*^2^ = 1.973, *df* = 2,* P* = 0.160	*χ*^2^ = 1.453, *df* = 2, *P* = 0.228
*Geographic area of residence*				
Central Israel	47	0 (0)	4 (8.5)	4 (8.3)
Jerusalem	16	0 (0)	3 (18.7)	3 (18.7)
Judean desert	60	1 (1.7)	10 (16.7)	11 (16.7)
Coastal plain	77	3 (3.9)	7 (9.1)	10 (9.1)
South-central Israel	8	0 (0)	0 (0)	0 (0)
Statistical significance			*χ*^2^ = 4.279, *df* = 4,* P* = 0.370	*χ*^2^ = 3.855, *df* = 4, *P* = 0.426

*Statistically significant association

^a^Statistical significance shown is based on serology results only

^b^Statistical significance shown is based on combined serology and PCR results

**Table 2:** Prevalence of exposure and infection with *B. persica* and demographic characteristics in 103 cats as determined by DNA detection by PCR and antibody detection by ELISA

Variable	Number of cats	PCR+ (%)	Seropositive (%)^a^	PCR+ or seropositive (%)^b^
Total	103	3 (2.9)	18 (17.5%)	20 (19.4%)
*Sex*				
Female	56	1 (1.8)	6 (10.7)	7 (12.5)
Male	47	2 (4.3)	12 (25.5)	13 (27.65)
Statistical significance			χ^2^ = 2.931, *df* = 1,* P* = 0.087	χ^2^ = 2.847, *df* = 1, *P* = 0.092
*Breed*				
Defined	11	0 (0)	4 (36.4)	4 (36.4)
Mixed	92	3 (3.3)	14 (15.2)	16 (17.4)
Statistical significance			χ^2^ = 1.757, *df* = 1,* P* = 0.185	χ^2^ = 1.210, *df* = 1, *P* = 0.271
*Age*				
Juvenile	11	0 (0)	0(0)	0 (0)
Adult	92	3 (3.2)	18 (19.5)	20 (21.5)
Statistical significance			χ^2^ = 1.528, *df* = 1,* P* = 0.232	χ^2^ = 1.471, *df* = 1, *P* = 0.225
*Antibiotic treatment*				
Yes	3	0 (0)	1 (33.3)	1 (33.3)
No	58	2 (3.9)	15 (25.8)	16 (15.7)
Unknown	42	1(2)	2 (4.76)	3 (18.4)
Statistical significance			*χ^2^ = 8.059,* df* = 2,* P* = 0.018	*χ^2^(2) = 6.889,* P* = 0.032
*Habitat*				
Urban	85	0 (0)	15 (17.6)	15 (17.6)
Rural	18	3 (16.6)	3 (16.6)	5(27.8)
Statistical significance			χ^2^ = 0.0001, *df* = 1,* P* = 1	χ^2^ = 0.434, *df* = 1, *P* = 0.510
*Climate*				
Mediterranean	70	2 (2.8)	11 (15.7)	12 (17.1)
Semi-arid	31	0	7 (22.5)	7 (22.5)
Arid	2	1 (50)	0	1 (50)
Statistical significance			χ^2^ = 1.134, *df* = 2,* P* = 0.567	χ^2^ = 1.413, *df* = 2, *P* = 0.493
*Geographic area of residence*				
Central Israel	7	0 (0)	2 (28.6)	2 (28.6)
Jerusalem	42	1 (2.4)	5 (11.9)	5 (11.9)
Judean desert	29	0 (0)	7 (24.1)	7 (24.1)
Coastal plain	22	1 (4.5)	4 (18.2)	5 (22.7)
South-central Israel	3	1 (33.3)	0 (0)	1 (33.3)
Statistical significance			χ^2^ = 3.037, *df* = 4,* P* = 0.552	χ^2^ = 2.828, *df* = 4, *P* = 0.587

*Statistically significant association

^a^Statistical significance shown is based on serology results only

^b^Statistical significance shown is based on combined serology and PCR results

There was no significant difference between the prevalence of PCR-positivity between the dog and cat populations (*χ*^2^ = 0.022, *df* = 1, *P* = 0.883), and no significant difference between the prevalence of ELISA-positivity between the dog and cat populations (*χ*^2^ = 1.602, *df* = 1, *P* = 0.206). The ratio between PCR-positivity and seropositivity in the dog population and the cat population, respectively, was 1:6. There was a significant difference between the prevalence of dogs that were positive by PCR (4/208) and those positive by serology (24/208) (*χ*^2^ = 13.823, *df* = 1, *P* = 0.0001). However, no significant difference was found between the prevalence of cats that were positive by PCR (3/103) and those positive by serology (18/103) (*χ*^2^ = 0.206, *df* = 1, *P* = 0.650).

Altogether, 13.5% (28/208) of the dogs and 19.4% (20/103) of the cats were positive for *B. persica* by serology combined with PCR, and there was also no significant difference in the overall prevalence between dogs and cats (*χ*^2^ = 1.444, *df* = 1, *P* = 0.230).

### Molecular and serological test results from the canine population

All four PCR-positive dogs were adults, of which three were females and three were purebred. All PCR-positive dogs resided in urban habitats, with three from an area with a Mediterranean climate and the fourth from a semi-arid area. Three of the PCR-positive dogs were not treated with antibiotic medications for at least 3 months prior to sampling, and data on the antibiotic treatment of the fourth dog were not available. Medical information was available for three of the PCR-positive dogs. None had suffered from bleeding, fever, icterus, anemia or thrombocytopenia, and the owners of one of the PCR-positive dogs reported lethargy and inappetence (Table 3). All four PCR-positive dogs were seronegative (OD < 0.23). PCR-positivity in these dogs was not found to be significantly associated with any of the tested variables; however, the number of PCR-positive dogs was too low to draw reliable conclusions.

**Table 3:** Prevalence of exposure and infection with *B. persica* and clinical characteristics in dogs as determined by PCR followed by DNA sequencing and antibody detection by ELISA

Variable	Number of dogs tested	PCR+ (%)	Seropositive (%)^a^	PCR+ or seropositive (%)^b^
*Fever*				
Yes	9	0 (0)	0 (0)	0 (0)
No	178	4 (2.2)	19 (10.7)	23 (12.9)
Total	187	4 (2.1)	19 (10.2)	23 (12.3)
Statistical significance			*χ*^2^ = 0.220, *df* = 1,* P* = 0.639	*χ*^2^ = 0.399, *df* = 1, *P* = 0.528
*Lethargy*				
Yes No Total	16 126 142	1 (6.3) 4 (3.1) 5 (3.5)	1 (6.3) 14 (1.1) 15 (10.6)	1 (6.3) 18 (14.3) 19 (13.3)
Statistical significance			*χ*^2^ = 0.027, *df* = 1,* P* = 0.870	*χ*^2^ = 0.250, *df* = 1, *P* = 0.617
*Bleeding*				
Yes No Total	10 186 196	0 (0) 4 (2.15) 4 (2)	1 (10) 19 (10.2) 20 (10.2)	1 (10) 23 (12.3) 24 (10.2)
Statistical significance			*χ*^2^ = 0.0001, *df* = 1,* P* = 1	*χ*^2^ = 0.0001, *df* = 1, *P* = 1
* Inappetence*				
Yes No Total	22 173 195	1 (4.5) 3 (1.7) 4 (2)	1 (4.5) 19 (11) 20 (10.3)	2 (0.9) 22 (12.9) 24 (12.3)
Statistical significance			*χ*^2^ = 0.318, *df* = 1,* P *= 0.573	χ^2^ = 0.020, *df* = 1, *P* = 0.886
*Icterus*				
Yes No Total	3 186 189	0 (0) 4 (2.1) 4 (2.1)	0 (0) 20 (10.7) 20 (10.6)	0 (0) 24 (12.9) 24 (12.7)
Statistical significance			*χ*^2^ = 0.0001, *df* = 1,* P* = 1	χ^2^ = 0.0001, *df* = 1, *P* = 1
*Anemia*				
Yes No Total	10 185 195	0 (0) 3 (1.6) 3 (1.5)	1 (10) 19 (10.3) 20 (10.3)	1 (10) 22 (11.9) 23 (11.8)
Statistical significance			*χ*^2^ = 0.0001, *df* = 1,* P* = 1	χ^2^ = 0.0001, *df* = 1, *P* = 1
*Thrombocytopenia*				
Yes No Total	9 191 200	0 (0) 3 (1.5) 3 (1.5)	4 (44.4) 16 (8.4) 20 (10)	4 (44.4) 19 (9.9) 23 (11.5)
Statistical significance			*χ^2^ = 8.739,* df* = 1,* P* = 0.003	*χ^2^ = 6.946,* df* = 1,* P* = 0.008

*Statistical significance association found between the variable above and a positive test result

^a^Statistical significance shown is based on serology results only

^b^Statistical significance shown is based on combined serology and PCR results

None of the demographic variables had a significant effect on seropositivity, and only the absence of antibiotic treatment in the previous 3 months was significantly associated with seropositivity (*χ*^2^ = 8.533, *df* = 2, *P* = 0.014) (Table 1). When evaluating the serological results for the dogs and their clinical variables (Table 3), only the presence of thrombocytopenia was found to have a significant association with seropositivity (*χ*^2^ = 8.739, *df* = 1, *P* = 0.003) and also with the combined prevalence of seropositivity or PCR-positivity (*χ*^2^ = 6.946, *df* = 1, *P* = 0.008) in these dogs.

### Molecular and serological test results for the feline population

All three PCR-positive cats were adults, of which one was female and two were males (Table 2). All three cats were from rural localities, with two from an area with a Mediterranean climate and the third cat from an arid area. Medical records for two of the PCR-positive cats were not available. The blood results from the third cat revealed regenerative anemia and thrombocytopenia, confirmed by visualization of a stained a blood smear. Of these three PCR-positive cats, one was found to be seropositive and two were seronegative.

None of the demographic variables had a significant effect on cat seropositivity, and only the absence of antibiotic treatment in the previous 3 months was significantly associated with seropositivity (*χ*^2^ = 8.059, *df* = 2, *P* = 0.018) (Table 2). When evaluating the cats' serological results and their clinical variables (Table 4), the presence of anemia was found to be significantly associated with seropositivity (*χ*^2^ = 14.808, *df* = 1, *P* = 0.0001) and with the combined prevalence of seropositivity or PCR-positivity (*χ*^2^ = 22.321, *df* = 1, *P* = 0.0001), and thrombocytopenia was associated with the combined prevalence of seropositivity or PCR-positivity (*χ*^2^ = 5.261, *df* = 1, *P* = 0.022).

**Table 4:** Prevalence of exposure and infection with *B. persica* and clinical characteristics in cats as determined by PCR followed by DNA sequencing and antibody detection by ELISA

Variable	Number of cats tested	PCR+ (%)	Seropositive (%)^a^	PCR+ or seropositive (%)^b^
*Fever*				
Yes No Total	5 71 76	0 (0) 1 (2.8) 1 (2.6)	1 (20) 12 (16.9) 13 (17.1)	1 (20) 13 (18) 14 (18.4)
Statistical significance			*χ*^2^ = 0.0001, *df* = 1,* P* = 1	*χ*^2^ = 0.0001, *df* = 1, *P* = 1
*Lethargy*				
Yes No Total	7 51 58	0 (0) 1(2) 1 (1.7)	2 (28.6) 9 (17.6) 11 (19)	2 (28.6) 10 (19.6) 12 (20.7)
Statistical significance			*χ*^2^ = 0.031, *df* = 1,* P* = 0.859	*χ*^2^ = 0.003, *df* = 1, *P* = 0.959
*Bleeding*				
Yes No Total	2 86 88	0 (0) 1 (1.1) 1 (1.1)	0 (0) 14 (16) 14 (15.7)	0 (0) 15 (17.4) 15 (17)
Statistical significance			*χ*^2^ = 0.0001, *df* = 1, P = 1	*χ*^2^ = 0.0001, *df* = 1, *P* = 1
*Inappetence*				
Yes No Total	14 74 88	0 (0) 1 (1.4) 1 (1.1)	3 (21.4) 11 (14.8) 14 (15.9)	3 (21.4) 12 (16.2) 15 (17)
Statistical significance			χ^2^ = 0.047, *df* = 1,* P* = 0.828	*χ*^2^ = 0.008, *df* = 1, *P* = 0.930
*Icterus*				
Yes No Total	4 84 88	0 (0) 2 (2.4) 2 (2.3)	1 (25) 12 (14.3) 13 (14.8)	1 (25) 13 (15.4) 14 (15.9)
Statistical significance			*χ*^2^ = 0.0001, *df* = 1,* P* = 1	*χ*^2^ = 0.0001, *df* = 1, *P* = 1
*Anemia*				
Yes No Total	7 81 88	1 (14.3) 1 (1.2) 2 (2.3)	5 (71.4) 8 (9.9) 13 (14.8)	6 (85.7) 8 (9.9) 14 (15.9)
Statistical significance			*χ^2^ = 14.808,* df* = 1, P = 0.0001	*χ^2^ = 22.321,* df* = 1,* P*
*Thrombocytopenia*				
Yes No Total	2 85 87	1 (50) 0 (0) 1 (1.1)	1 (50) 12 (14.1) 13 (14.9)	2 (100) 12 (14.1) 13 (14.9)
Statistical significance			*χ*^2^ = 0.163, *df* = 1,* P* = 0.686	*χ^2^ = 5.261,* df* = 1, P = 0.022

*Statistical significance association found between the variable above and a positive test result

^a^Statistical significance shown is based on serology results only

^b^Statistical significance shown is based on combined serology and PCR results

### Relationship between positivity for *B. persica* and clinical findings

Of the 195 dogs with medical records, 21.5% (40/195) presented with at least one of the borreliosis-associated clinical findings, including fever, lethargy, bleeding, inappetence, icterus, anemia and thrombocytopenia, while only 15% (6/40) were positive by serology or PCR for *B. persica*. Of the 87 cats with medical records, 23% (20/87) had at least one clinical sign compatible with borreliosis, of which seven were positive by serology or PCR for *B. persica*.

Overall, of the 282 animals for which accessible medical data were available, 21.3% (60/282) had at least one clinical finding that could be associated with borreliosis. Thirteen of the 60 animals (21.7%) with clinical signs possibly associated with the disease were positive for *B. persica* infection by either serology or PCR. As already mentioned, the clinical findings that were significantly associated with seropositivity for *B. persica* were thrombocytopenia in dogs and anemia in cats, with thrombocytopenia also associated with the combined prevalence of seropositivity or PCR-positivity in felines.

## Discussion

When disease caused by *B. persica* was described in cats and dogs for the first time in Israel, in a study from 2016, it was considered to be a rare infection, with five clinical cases in cats and five in dogs [10]. These 10 cases were recorded over a 12-year period in Israel, and one case report of a dog originated in Iran [10, 21]. The current study retrospectively surveyed samples from cats and dogs brought for medical treatment and not suspected of relapsing fever borreliosis during their medical workup. The results of nearly 2% and 3% of a spirochetemia positivity in the canine and feline population, respectively, detected by PCR at the time of admission, and 11.5% and 17.5% seropositivity in dogs and cats, respectively, indicate that infection with *B. persica* is more widespread in companion animals in Israel than previously estimated. As this is also a human disease with a wildlife animal reservoir, the role of domestic cats and dogs as carriers of this infection should be considered.

Epidemiological studies on relapsing fever *Borrelia* spp. infections in pet animals are scarce. The present study is the first serological study on any relapsing fever *Borrelia* spp. in cats and the first survey to combine serology and PCR on relapsing fever *Borrelia* in dogs and cats. The only PCR survey on relapsing fever *Borrelia* infection in cats was a study which detected *B. miyamotoi* DNA in two of 49 healthy cats in Maryland in the USA [17]. *Borrelia hispanica* is the only other relapsing fever-inducing spirochete reported to cause clinical disease in the cat [16], except for *B. persica*. No detailed clinical and demographic survey on relapsing fever borreliosis in cats has been published to date. Several relapsing fever *Borrelia* spp. are known to infect dogs in different areas of the world; however, only two large-scale surveys of relapsing fever in dogs have been published, both of which describe *B. turicatae* infection of dogs from Texas (USA) [22, 23]. In the first of these studies, the seroprevalence of *B. turicatae* was studied in 878 domestic dogs in Texas and evaluated by recombinant glycerophosphodiester phosphodiesterase (GlpQ) antigen immunoblot, with the results showing 1.99% seroprevalence [22]. In the second study, which used PCR technology, 0.68% of 1171 dogs whose blood samples were submitted to a veterinary laboratory for testing of various conditions were positive by PCR for *B. turicatae. Borrelia turicatae* infections were detected in several ecoregions of Texas and the regional prevalence in areas where positive dogs were detected ranged from 0.85 to 1.65% [23]. Although *B. turicatae* infection has been reported to cause disease with clinical signs comparable to a *B. persica* infection in dogs [13, 14, 24], no detailed clinical data were available for the infected dogs included in these surveys from Texas.

In the present study from Israel, infection in cats and dogs was prevalent in both sexes, all ages, different animal breeds, urban and rural settings, Mediterranean and semi-arid climates and all five geographic areas of residence included in the study. The geographic distribution of infection is in agreement with that reported in other studies on *B. persica* infection in ticks, wildlife and humans in Israel, which describe infection in almost all areas of the country, except for the very southern part close to the Red Sea [2, 12, 25, 26]. Urban and rural settings in Israel often provide suitable conditions for the survival *O. tholozani*, the argasid tick vector of *B. persica*; these include shady areas characterized by high humidity and mild temperature [4]. It is therefore not surprising that pet animals from both settings are infected with this pathogen.

Of the clinical abnormalities reported previously in dogs and cats with relapsing fever [10, 13–16, 24], thromobocytopenia in dogs and anemia in cats emerged in our study as being significantly associated with seropositivity for *B. persica* infection, and thrombocytopenia in cats was significantly associated with the combined prevalence of seropositivity or PCR-positivity. The absence of other clinical findings might be due to the limited number of animals evaluated in the study or because seropositivity might still be detected long after the initial infection and the resolution of possible clinical disease. Both anemia and thrombocytopenia are characteristic findings in bacterial and protozoal tick-borne diseases of dogs and cats and have been reported in the majority of the feline and canine *B. persica* cases included in the initial description of the disease in these animals in Israel [10]. Anemia was also reported in a cat with *B. hispanica* infection from Spain [16], and thrombocytopenia was reported from dogs infected with *B. turicatae* in the USA and from a single case of *B. hermsii* infected dog from the USA [14, 15, 24].

In contrast to the detection of *B. persica* by PCR by direct amplification of the spirochetes' DNA from the blood sample and verification by DNA sequencing, detection of antibodies reactive with *B. persica* antigens could be less specific as cross-reactivity with antigens of other organisms may occur. Because Israel and its surrounding region in the Middle East are not endemic for Lyme borreliosis (LB) and no autochthonous infections with the LB group borreliae or other relapsing fever borreliae have been reported in humans, dogs and cats in Israel, serological cross-reactivity with a closely related borrelial agent is not likely.

The finding that all PCR-positive dogs and two of three PCR-positive cats were seronegative for *B. persica* could be explained by the possibility that these animals were in the early stages of the infection, before specific antibodies were produced and detectable in the animals' sera. Another option is that infection in some cases may not be associated with a strong or long-lasting antibody production and that perhaps animals remain carriers of *B. persica* infection without having detectable antibodies against it. The length of time during which antibodies against *B. persica* persist in the animal following acute infection is currently unknown and, therefore, it is not possible to assess the duration of time between infection and the onset of detectable antibodies in the blood serum. It may also be possible that animals could be seropositive while *B. persica* infection persists in other organs of their bodies and not in the peripheral blood, resulting in a negative PCR result for blood samples. In a study of natural infection in rock hyraxes (*Procavia capensis*) with *B. persica* in Israel, in which blood as well as spleen samples were tested by PCR, of the five hyraxes that tested positive for the spleen samples, only two were also PCR-positive for the blood samples, with blood samples from the other three hyraxes PCR-negative [11]. In an experimental study with mice infected intradermally with *B. persica*, the brain and skin were found to be major target organs for spirochete dissemination, and *B. persica* was detected by PCR and cultured from the brains of the majority of infected mice at day 50 post-infection, even in the absence of blood spirochetemia [27]. These findings from natural and experimental infections in other species of animals indicate that *B. persica* infection disseminates to several organs and can be found in animals with a negative blood PCR. This may support the assumption that some PCR-negative, yet seropositive dogs and cats are temporarily or persistently infected with *B. persica* organisms, which occasionally may spread back to the major blood circulation, while other dogs and cats clear the infection and remain seropositive with decaying levels of antibodies for variable periods of time. The ratio of 1:6 between PCR-positivity and seropositivity found for both cats and dogs in this study may suggest that similar dynamics between blood spirochetemia and seropositivity exists in both species of companion animals.

The use of antibiotic medications from 3 months prior to sample collection was recorded in the studied animals since *B. persica* is sensitive to several antibiotics, possibly leading to negative PCR results, if animals were treated recently [10, 28, 29]. Both the dogs and cats that had received antibiotics close to the time of sampling showed significantly lower seropositivity rates and also lower combined seropositivity or PCR-positivity rates than untreated animals infected with *B. persica*, probably reflecting the spirochete’s sensitivity to antibiotics. In different studies, it was shown that even one preventive treatment with doxycycline after a tick bite was sufficient to prevent disease in humans [29] and that the disappearance of spirochetemia in a treated cat was evident 1 day after initial treatment with ciprofloxacin [10].

The moderate infection rates in cats and dogs with *B. persica* detected in this study and the infection rates with *B. turicatae* reported from dogs in Texas [23] warrant that relapsing fever *Borrelia* spp. be included as one of the pathogens that canine and feline blood donors should be screened for in Israel and other endemic areas for these infections. Transfusion transmission of several relapsing fever *Borrelia* spp. has been reported in humans and also experimentally in laboratory rodents and is thus likely possible also in dogs and cats [30, 31]. In addition, cats and dogs may serve as sentinels for the risk of human *B. persica* infection in the same location or region as they are exposed to tick bites and pathogen transmission and live in close proximity to humans.

This study had several limitations, which include a relatively limited number of cats and dogs that were surveyed and missing information on some of the clinical cases. No information was available on other conditions that may have affected the animals' clinical data, including on co-infections with agents such as the feline immunodeficiency virus (FIV), feline leukemia virus (FeLV), *Ehrlichia* spp. and *Babesia* spp. The participating clinics did not screen for these infections in the majority of the animals included in the study. This study included mostly animals from the center of Israel where the majority of the human population is located, and not from the most northern and southern parts of the country. Despite these limitations, this study reveals important information on *B. persica* infection and demonstrates that this infection is widely spread in the feline and canine populations of the surveyed areas.

## Conclusions

*Borrelia persica* infection detected by molecular and serological assays is more prevalent and widespread in cat and dog populations than previously thought and appears to be underreported in Israel. Dogs and cats may play a role as reservoirs and sentinels for human infection, and care should be taken to prevent transfusion transmission of the disease between blood donor and recipient animals.

## Data Availability

All data generated or analyzed during this study are included in this published article.

## Abbreviation list

ELISA: Enzyme-linked immunosorbent assay
TBRF: Tick-borne relapsing fever
